# Molecular cytogenetic studies of a male carrier with a unique (Y;14) translocation: Case report

**DOI:** 10.1002/jcla.23614

**Published:** 2020-10-14

**Authors:** Shuang Chen, Qi Xi, Xinyue Zhang, Yuting Jiang, Leilei Li, Ruizhi Liu, Hongguo Zhang

**Affiliations:** ^1^ Center for Reproductive Medicine and Center for Prenatal Diagnosis First Hospital Jilin University Changchun China

**Keywords:** cryptozoospermia, genetic counseling, male infertility, Y;14 translocation

## Abstract

**Background:**

Chromosome translocation is a genetic factor associated with male infertility. However, cases of Y chromosome/autosome translocation are rare. Individuals with translocation between the Y chromosome and an autosome have a variety of different clinical phenotypes. There is a need for further study of molecular cytogenetic feature of those with Y chromosome translocation.

**Methods:**

We reported that an apparently healthy 31‐year‐old man, 168 cm tall and weighing 65 kg, had a 2‐year history of primary infertility after marriage. Clinical diagnostic techniques included semen analysis, hormone measurements, cytogenetic analysis, fluorescence in situ hybridization (FISH), and high‐throughput multiplex ligation‐dependent probe amplification semiconductor sequencing. Detailed genetic counseling was provided to the patient. Intracytoplasmic sperm injection treatment combined with preimplantation genetic diagnosis was chosen with the aim of achieving a successful pregnancy.

**Results:**

Semen analysis revealed cryptozoospermia. Hormone levels were within the normal limits. Sequencing results indicated the presence of the sex‐determining region on Yp, and AZFa, AZFb, and AZFc regions on Yq. The patient's karyotype was 45,X,psu,dic(Y;14)(p11.3;q11.2), which was confirmed by cytogenetic analysis and FISH.

**Conclusion:**

This study reports a case of cryptozoospermia in a male patient with a Y;14 chromosomal translocation. When clinical karyotyping has revealed potential Y chromosome abnormality, FISH or molecular detection should be further performed to facilitate identification of the chromosomal breakpoint.

## 1 INTRODUCTION

In humans, the incidence of Y‐autosome chromosomal abnormalities is about 1/2000 live births.^1^ Cases of Y‐autosome translocations are rare, but may be identified in both fertile and sterile males.^2^ They are closely related to male infertility, resulting in azoospermia, severe oligozoospermia, or asthenospermia in the carrier males.^3^, ^4^, ^5^ Some carriers may also have normal semen parameters and produce offspring without phenotypic repercussions.^6^ A review of those with Y‐autosome translocation indicated that most translocations lead to azoospermia or oligozoospermia.^2^ These conditions may be related to breakpoints, balanced, or unbalanced chromosomal changes involved in translocation.^7^, ^8^ Previous studies were mainly based on G‐banding analysis,^2^ which could not determine detailed structures.^5^ Therefore, some unique chromosomal abnormalities require further identification of breakpoints or detailed structures. Here, we describe a male carrier with a unique (Y;14) translocation, including his molecular cytogenetic characteristics.

## 2 MATERIALS AND METHODS

This study was approved by the Ethics Committee of the First Hospital of Jilin University (No. 2019‐230). Written informed consent was obtained from the patient for publication of this case report.

### 2.1 Semen analysis

Semen samples were collected by masturbation after abstinence of 3‐5 days. After these samples had been completely liquefied at 37°C, semen analysis was performed in accordance with the World Health Organization's standard protocol.^9^ If no sperm were found, semen samples were centrifuged (3000 *g* for 15 minutes) and the sediment was then reexamined. The patient was confirmed to have cryptozoospermia by the analysis of two centrifuged semen samples at an interval of 1 month.

### 2.2 Detection of reproductive hormones

Serum reproductive hormones (follicle‐stimulating hormone [FSH], luteinizing hormone [LH], prolactin [PRL], estradiol [E_2_], and testosterone [T]) were determined using a commercially available kit and the Elecsys 2010 chemistry analyzer (Roche).

### 2.3 Cytogenetic analysis

After the provision of informed consent, peripheral blood was collected from the patient and lymphocytes were cultured for 72 hours in appropriate culture medium (Cell Preservation Medium; Sinochrome). Karyotype analysis was performed using G‐band staining in accordance with standard cytogenetic procedures. Twenty metaphases were analyzed for this sample.

### 2.4 Molecular analysis

To analyze the frequency of Y chromosome microdeletions, human blood samples were collected from the patient, from which genomic DNA was extracted. Semiconductor sequencing was performed in accordance with our previous study.^10^ Markers included 36 sequence‐tagged sites, the sex‐determining region on the Y chromosome (SRY), and zinc finger proteins, namely X‐linked and Y‐linked zinc finger proteins used as internal controls, as described in our previous study.^5^

### 2.5 Fluorescence in situ hybridization (FISH) analysis

FISH was performed to detect the sex‐determining region on the Y chromosome (SRY) and the long arm of the Y chromosome (Yq12). The detailed operating protocol was followed as in our previous study.^11^

## 3 CASE INTRODUCTION

**Figure 1:**
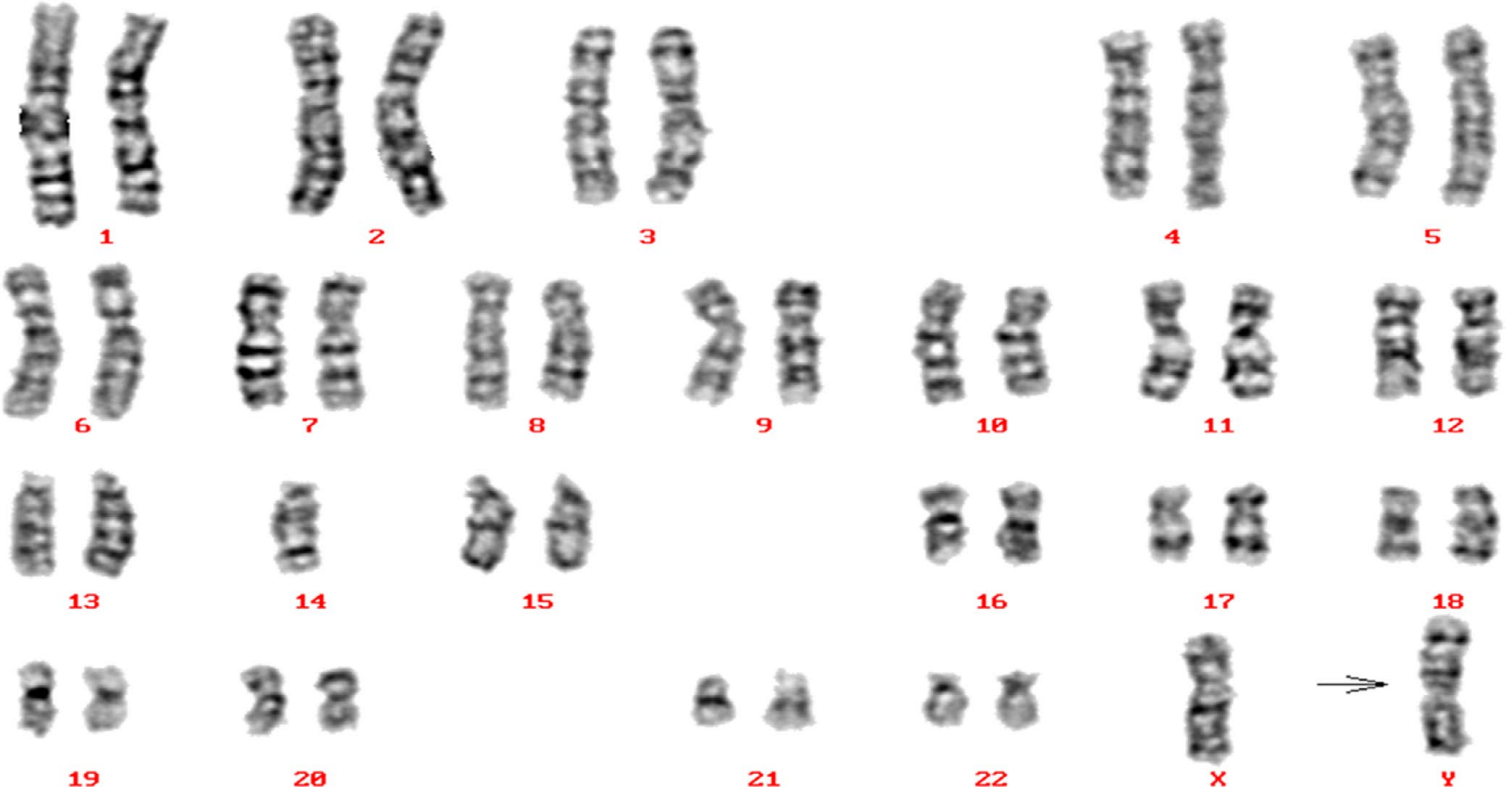
The karyotype of the case was found to involve 45,X,t(Y;14)

**Figure 2:**
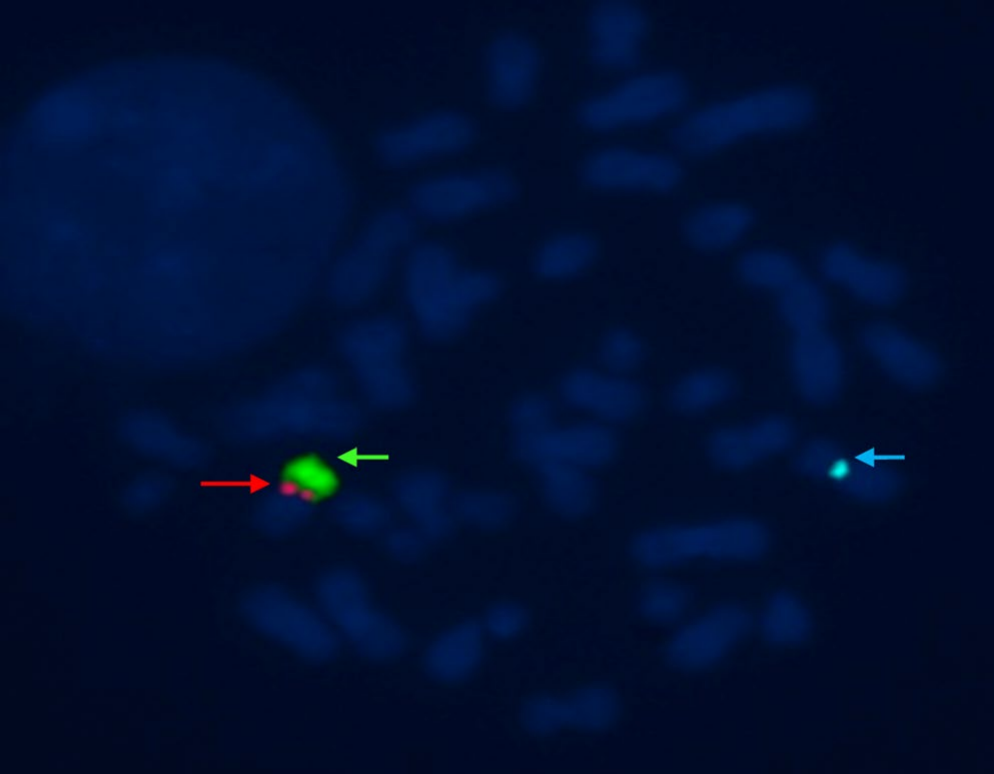
Metaphase‐FISH results of an alphoid probe for SRY and Yq1 probe 2: the red arrow indicates the SRY signal, the green arrow Yq12, and the blue arrow the X centromere signal

A 31‐year‐old male had a 2‐year history of infertility after marriage and consulted at an andrological clinic. The patient was 168 cm tall and 65 kg in weight. He exhibited a well‐developed male phenotype. Physical examination showed a normal male habitus. Physical examinations were performed by an urologist to detect varicocele and to measure testis volume using a Prader orchidometer (Pro‐Health). The left and right testes were each approximately 12 mL in volume. The patient did not present pathological varicocele. Routine semen analysis revealed azoospermia and sperm were occasionally seen after centrifugation of the semen. The patient was thus diagnosed with cryptozoospermia. Hormone analysis showed that serum FSH, LH, E_2_, PRL, and T levels were within the normal limits. Cytogenetic analysis showed that the patient had unbalanced Y;14 chromosome translocations, but the breakpoint could not be determined (Figure 1). Further molecular analysis showed the presence of the SRY gene and that 36 loci in the AZF region of the Y chromosome had not been deleted. Because SRY exists on the short arm of the Y chromosome, while the AZF gene exists on the long arm of Y chromosome, we performed FISH detection. Both SRY and Yq12 probes were on chromosomes derived from Y and 14 (Figure 2). Therefore, we speculated that the karyotype of the patient was 45,X,psu,dic(Y;14)(p11.3;q11.2). Detailed genetic counseling was performed for the patient. Despite the provision of information on the procedure, the patient's parents refused to undergo chromosomal analysis.

Following genetic counseling and informed consent, the patient has chosen to pursue intracytoplasmic sperm injection treatment combined with preimplantation genetic diagnosis. Eventually, a phenotypically normal boy with the same chromosome as his father is successfully delivered.

## 4 DISCUSSION

Chromosome translocation is one of the genetic factors known to be associated with male infertility.^12^ However, cases of Y chromosome/autosome translocation are rare.^13^ Individuals with translocation between the Y chromosome and an autosome have a variety of different clinical phenotypes. Aftab et al^3^ reported a rare case of de novo balanced reciprocal Y:1 chromosomal translocation presenting with azoospermia. Orrico et al^13^ reported a male carrier of an unbalanced Y;21 translocation showing azoospermia. Moreover, Gunel et al^14^ reported an individual with azoospermia and cryptorchidism who carried a de novo reciprocal t(Y;16) translocation. Our team also reported one male carrier of an unbalanced Y;22 translocation showing oligoasthenozoospermia.^5^ Furthermore, Morales et al^6^ reported a large family with pseudodicentric 22;Y translocation transmitted through four generations without phenotypic repercussions. In the present study, our case showed normal hormone levels and a male phenotype, normal testicular size, and the presence of the Yp and Yq regions. From the karyotype shown in Figure 1, the patient had unbalanced Y;14 chromosome translocations, but the breakpoint could not be determined. Semen analysis revealed that the patient displayed cryptozoospermia. These results suggest that certain translocations or chromosomal regions can be directly associated with spermatogenesis or maturation.

To analyze the breakpoint in detail, FISH detection was performed. Both SRY and Yq12 probes were on chromosomes derived from Y and 14. Therefore, the karyotype of 45,X,psu,dic(Y;14)(p11.3;q11.2) was diagnosed. Pasquier et al^15^ reported that karyotyping remains a powerful and cheap technology that is available globally and can successfully detect some chromosome rearrangements. In some unique cases, a chromosomal imbalance was identified by conventional karyotyping, FISH, or another molecular technology. In a review, Orrico et al^13^ revealed that fewer than 40 cases of Y‐autosome translocation have been reported in the literature; all of these differed with regard to the autosome and/or the involved regions of the Y chromosome. In our case, the patient with 45,X,psu,dic(Y;14)(p11.3;q11.2) presented with cryptozoospermia. Through assisted treatment of intracytoplasmic sperm injection treatment, the patient gave birth to his own offspring. These results suggest that the detailed diagnosis and assisted reproductive technology should be recommended for male carriers with Y chromosome abnormalities.

A limitation of this study is the lack of detection of the origin of this chromosomal abnormality because the patient's parents refused to undergo chromosomal analysis.

## 5 CONCLUSIONS

We report a case of cryptozoospermia in a male patient with a Y;14 chromosomal translocation. When clinical karyotyping has revealed potential Y chromosome abnormality, FISH or molecular detection should be further performed to facilitate identification of the chromosomal breakpoint.
